# Prevalence of micronutrient deficiencies across diverse environments in rural Madagascar

**DOI:** 10.3389/fnut.2024.1389080

**Published:** 2024-05-16

**Authors:** Christopher D. Golden, Jessica Zamborain-Mason, Alexander Levis, Benjamin L. Rice, Lindsay H. Allen, Daniela Hampel, James Hazen, C. Jessica E. Metcalf, Hervet J. Randriamady, Setareh Shahab-Ferdows, Stephanie M. Wu, Sebastien Haneuse

**Affiliations:** ^1^Department of Nutrition, Harvard TH Chan School of Public Health, Boston, MA, United States; ^2^Department of Environmental Health, Harvard TH Chan School of Public Health, Boston, MA, United States; ^3^Madagascar Health and Environmental Research (MAHERY), Maroantsetra, Madagascar; ^4^Department of Biostatistics, Harvard TH Chan School of Public Health, Boston, MA, United States; ^5^Department of Ecology and Evolutionary Biology, Princeton University, Princeton, NJ, United States; ^6^Western Human Nutrition Research Center, Agricultural Research Service (USDA), Davis, CA, United States; ^7^Department of Nutrition, College of Agricultural and Environmental Sciences, University of California, Davis, Davis, CA, United States; ^8^Catholic Relief Services, Baltimore, MD, United States

**Keywords:** micronutrient deficiencies, food security, iron deficiency, vitamin deficiency, zinc deficiency, inflammation, vitamin A, vitamin B_12_

## Abstract

It is estimated that billions of people around the world are affected by micronutrient deficiencies. Madagascar is considered to be particularly nutritionally vulnerable, with nearly half of the population stunted, and parts of the country facing emergency, near famine-like conditions (IPC4). Although Madagascar is generally considered among the most undernourished of countries, empirical data in the form of biological samples to validate these claims are extremely limited. Our research drew data from three studies conducted between 2013–2020 and provided comprehensive biomarker profile information for 4,710 individuals from 30 communities in five different ecological regions during at least one time-point. Estimated prevalences of nutrient deficiencies and inflammation across various regions of rural Madagascar were of concern for both sexes and across all ages, with 66.5% of the population estimated to be deficient in zinc, 15.6% depleted in vitamin B_12_ (3.6% deficient), 11.6% deficient in retinol, and lower levels of iron deficiency (as indicated by 11.7% deficient in ferritin and 2.3% deficient assessed by soluble transferrin receptors). Beyond nutrient status biomarkers, nearly one quarter of the population (24.0%) exhibited chronic inflammation based on high values of α-1-acid glycoprotein, and 12.3% exhibited acute inflammation based on high values of C-reactive protein. There is an 8-fold difference between the lowest and highest regional observed prevalence of vitamin B_12_ deficiency, a 10-fold difference in vitamin A deficiency (based on retinol), and a 2-fold difference in acute inflammation (CRP) and deficiencies of zinc and iron (based on ferritin), highlighting strong geographical variations in micronutrient deficiencies across Madagascar.

## Introduction

Globally, it is estimated through biomarker assessment that 372 million pre-school aged children and 1.2 billion women of reproductive age are affected by deficiencies of iron, zinc, and/or vitamin A (1). Other biomarkers are less frequently assessed though deficiencies may be equally prevalent. These deficiencies are deeply concerning as some regions have experienced global declines in dietary micronutrient density (e.g., sub-Saharan Africa) over the past 50 years (2). It is critical for public health and nutritional targeting to identify geographies and populations vulnerable to nutrient deficiencies.

At population scales, biomarkers are used to classify the prevalence of micronutrient deficiencies. These prevalences can vary widely across nations, within nations, and even within households. International and subnational variation could be attributed to variability in ecologies (3), economies (4), food systems (5), and landscapes of disease (6).

Although Madagascar is generally considered among the most undernourished of countries, empirical data in the form of biological samples to validate these claims are extremely limited: one study consisting of approximately 2,000 women of reproductive age found moderate levels of iodine deficiency (7), while a recent trial of lipid-based nutritional supplements in fewer than 400 children documented a high prevalence of iron deficiency, a moderate prevalence of vitamin A deficiency, and moderate to high prevalence of inflammation status (8).

To address this gap, as well as to inform targeted food-based programming efforts and spatial prioritization of public health interventions, we: (1) provide prevalence estimates of five micronutrient deficiencies (two iron-related markers, zinc, and vitamins A and B_12_) and two inflammation markers in Malagasy people; and (2) quantify regional patterns of micronutrient deficiencies and inflammation status across diverse environments and food systems. We do not analyze the proximate drivers of these micronutrient deficiencies here because it is beyond the scope of the current research. The primary objective of this study is to highlight the geographical variation in micronutrient deficiencies to highlight regions in need of food system and nutritional interventions, allowing for targeting of particular micronutrient deficiencies and subpopulation most at risk.

## Methods

### Study population

**Figure 1:**
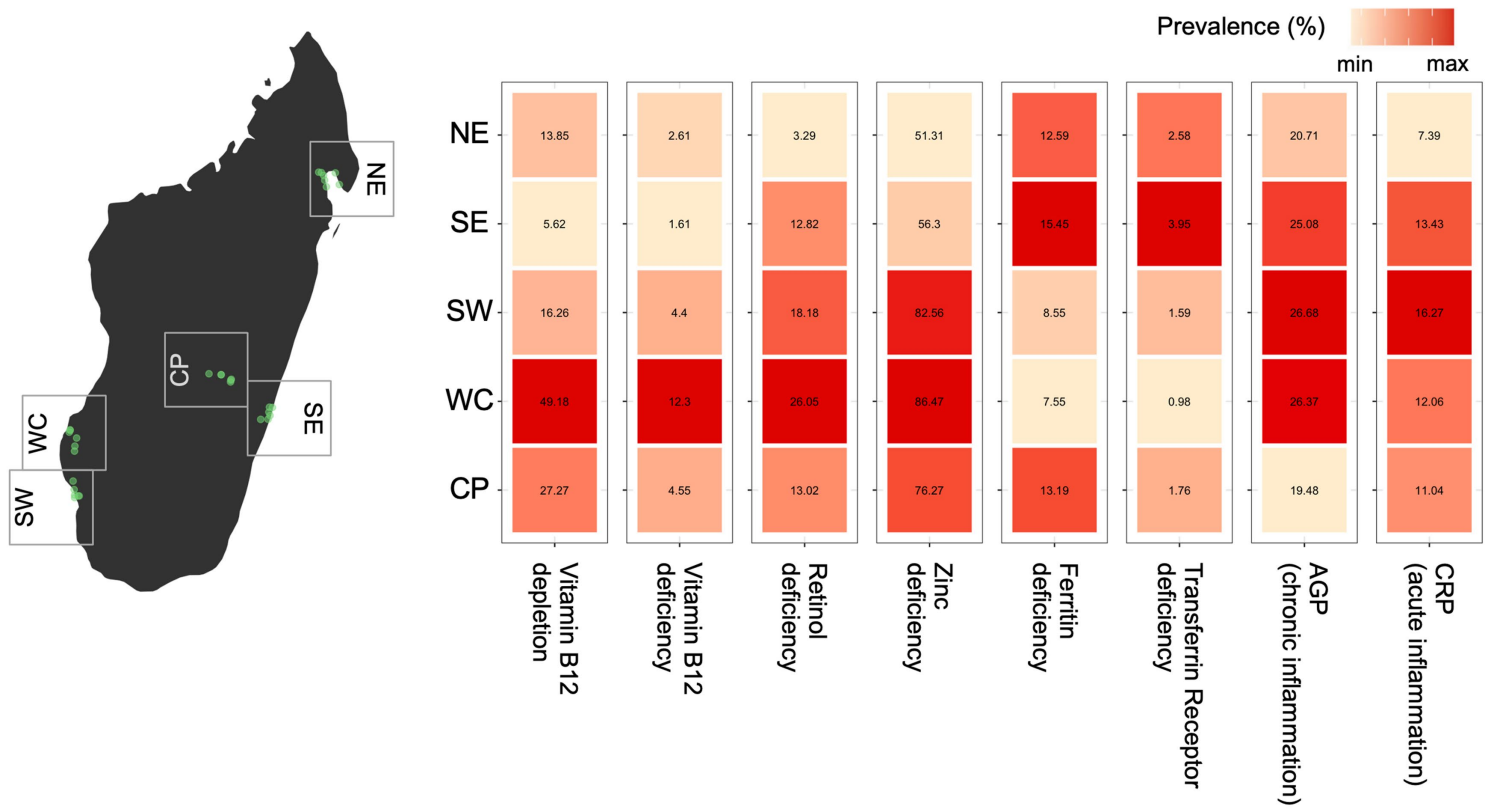
Observed variation in micronutrient deficiency and inflammation prevalence for all ages and sexes across regions of Madagascar (using the first sample for each individual when multiple samples were collected). For each biomarker, the relative difference in prevalence is shown as a heatmap with the color scale ranging from the minimum to maximum observed prevalence by region. The prevalence (as a percentage) estimate is shown within each cell.

A total of 7,493 samples were collected from 4,710 participants in two prospective cohort studies (9, 10) and one cross-sectional study (11), targeting rural communities across Madagascar (Table 1). The studies comprised 30 communities across diverse social, economic, and ecological zones, clustered in five regions throughout the country: northeastern rainforest (NE), southeastern rainforest (SE), southwestern spiny desert (SW), western coast scrubland (WC), and the central plateau (CP) (Figure 1). All three studies utilized a complete household census, with a total of 1,098 households randomly selected for inclusion. Subsequently, all individuals (within each selected household), regardless of age and sex, were enrolled and asked to provide comprehensive survey data and biological samples (fecal, fingernail, whole blood spots, and venous blood plasma). No exclusion criteria other than being too physically ill to participate were considered. All samples were taken from fasted individuals and cold chain was maintained between the field and laboratory, staying in liquid nitrogen containers until being transferred to dry ice shipments to the United States where they were stored in −80°C freezers. Complete details for all three studies have been published elsewhere (9–11).

**Table 1:** Summary of locations, populations, and biomarkers sampled in Madagascar.

Region	# of Communities	# of Individuals sampled	Study time period	Sample time points	Biomarkers assessed	Ref.
Northeastern, Analanjirofo, Maroantsetra (NE)	2	593	2013–2014	3	Zinc, iron, ferritin, transferrin receptors, AGP, CRP, retinol, vitamin B_12_	(9)
Northeastern, Analanjirofo, Maroantsetra (NE)	5	745	2016	2	Zinc, iron, ferritin, transferrin receptors, AGP, CRP, retinol, vitamin B_12_, fatty acid profile	(10)
Southeastern, Vatovavy Fitovinany, Mananjary (SE)	6	986	2017	1	Zinc, iron, ferritin, transferrin receptors, AGP, CRP, retinol, vitamin B_12_, fatty acid profile	(11)
Southwestern, Atsimo Andrefana, Toliara (SW)	6	889	2017	1	Zinc, iron, ferritin, transferrin receptors, AGP, CRP, retinol, vitamin B_12_, fatty acid profile	(11)
Western, Atsimo Andrefana, Morombe (WC)	6	621	2017	1	Zinc, iron, ferritin, transferrin receptors, AGP, CRP, retinol, vitamin B_12_, fatty acid profile	(11)
Central Plateau, Amoron’i Mania, Fandriana (CP)	6	459	2017	1	Zinc, iron, ferritin, transferrin receptors, AGP, CRP, retinol, vitamin B_12_, fatty acid profile	(11)

### Laboratory analysis of biomarkers

Plasma vitamin B_12_ and ferritin were measured using the Cobas e411 analyzer (Roche Diagnostics, Indianapolis, IN, USA) according to the manufacturer’s protocol. C-reactive protein (CRP), α-1-acid glycoprotein (AGP), and soluble transferrin receptors (TfR) were measured using a COBAS® INTEGRA 400 plus multianalyte analyzer (Roche Diagnostics, Indianapolis, IN, USA). Retinol was measured by HPLC-MWL as previously described (12) using a 1,260 HPLC system (Agilent, Santa Clara, CA, USA) and tocol as internal standard (IS). UTAK pooled human plasma with known retinol values was used as quality control (QC) with every analytical run. Zinc concentrations were measured by NexION 2000 ICP-MS (Perkin Elmer, Waltham, MA, USA) after the addition of germanium as an Internal Standard and subsequent sample digestion (200 μL) using 1 N nitric acid (3.8 mL) overnight. Accuracy was monitored using Seronorm™ Trace Element Serum L-1 and L-2 (Sero, Billingstad, Norway), and the same UTAK plasma was used for QC purposes with every analytical run.

### Evaluating nutrient deficiencies and inflammatory status

We considered eight binary outcome variables: deficiency status for each of the five nutrient biomarkers (vitamin B_12_, retinol, zinc, ferritin, and transferrin receptors); depletion of vitamin B_12_, and two inflammatory status indicators based on CRP (a measure of acute inflammation) and AGP (a measure of chronic inflammation). Thresholds for determining nutrient deficiency and inflammatory status were based on international standards and validated clinical research (Table 2).

**Table 2:** Thresholds for deficiency assessments of nutritional biomarkers.

Biomarker	Threshold for deficiency	Reference
Vitamin B_12_	<148 pmol/L for deficiency; < 221 pmol/L for depletion	(13)
Retinol	< 0.70 μmol/L	(14)
Zinc	< 65 μg/dL for children less than 10 years < 74 μg/dL for males greater than 10 years < 70 μg/dL for females greater than 10 years	(15)
Ferritin	< 12 ng/mL for children less than 5 years < 15 ng/mL for individuals 5 years and older	(16)
Transferrin receptor	> 8.3 mg/L	Manufacturer recommendations (Ramco Laboratories Inc.), see (17)
C-reactive protein (CRP)	> 5 mg/L to indicate acute inflammation	(18)
Alpha(1)-acid glycoprotein (AGP)	> 1 g/L to indicate chronic inflammation	(18)

### Adjusting biomarkers for inflammation

Infection, injury, or other forms of chronic or acute inflammation can influence the measurement and interpretation of biomarker levels and corresponding deficiency status (18–20). For example, studies across different populations have found that retinol concentrations tend to be elevated under inflammation, while ferritin and transferrin levels are depressed (21, 22). Without accounting for these effects, micronutrient deficiency prevalences may be under- or over-estimated.

To account for these potential biases, we adjusted retinol, zinc, ferritin, and transferrin receptor levels based on findings from the Biomarker Reflecting Inflammation and Nutritional Determinants of Anemia (BRINDA) project (23, 24). Specifically, we employed a regression correction developed by Namaste et al. (25), consistently adjusting levels of retinol, zinc, ferritin, and transferrin receptor by AGP, CRP and malaria status, for all individuals except those in the lowest decile of AGP or CRP. Our subsequent analyses excluded nutritional biomarker measurements where AGP, CRP or malaria status were not available. Missing data were omitted from analysis.

### Statistical analysis

To characterize spatial variability in nutrient deficiencies and inflammation status while accounting for inherent data structures in our data (e.g., sex or age), we separately modeled each outcome using Bayesian multilevel logistic regression models. These models included random effects that acknowledge and represent the nested structure in the data: repeated measures across individuals over time, individuals within households, households within communities, and communities within regions (Supplementary materials), and fixed effects for season (rainy vs. dry), age, and sex, with age standardized by subtracting the mean and dividing by two times the standard deviation (26). By acknowledging the nested dependence structure inherent to the sampling design, the fit of the models enabled a quantitative decomposition of variation among various components. Each analysis used all available outcome data with missing measurements excluded (see Supplementary Table S1 for sample sizes that informed each model). Throughout, non-informative flat priors were adopted for fixed effects, and half Student-t priors with 3 degrees of freedom and scale parameter 2.5 for the random effect standard deviations (27). To assess sensitivity to the choice of priors and some individuals not having repeated samples, we reran analyses using scale parameters 0.5 and 5.5 in the prior for random effects, corresponding to more and less concentrated prior distributions. Our spatial variability results were robust to these sensitivity checks. Detailed methods are provided in the Supplementary materials. Here, we have purposely conducted this descriptive analysis of the geographical patterns of nutritional biomarkers accounting for potential confounders (e.g., sex, age or season; see Supplementary materials), and future research will investigate the specific dietary and infectious disease drivers of nutritional status.

All analyses were performed with RStan via the brms package in R (28, 29). For each model, four chains of 3,000 iterations were run, with 800 used as burn-in, and thinning to every third iteration of each chain. Convergence was assessed by inspecting the potential scale reduction factors for all parameters, and comparing it to the recommended nominal threshold of 1.05 (30). All models fit the data well (Supplementary materials).

## Results

### Estimating the prevalence of inflammation and micronutrient deficiencies based on biomarkers

The estimated prevalences of BRINDA-adjusted nutrient deficiencies across various regions of rural Madagascar were generally moderate to high in both sexes and across all ages, with 65.5% of the population estimated to be deficient in zinc, 15.6% depleted in vitamin B_12_, 11.6% deficient in retinol, and lower levels of iron deficiency (as indicated by 11.7% deficient in ferritin and 2.3% deficient in soluble transferrin receptor) (Table 3). Beyond nutrient biomarkers, nearly one quarter of the population (24.0%) exhibited chronic inflammation based on high values of AGP, and 12.3% of the population exhibited acute inflammation based on high values of CRP (Table 3).

**Table 3:** Summary of overall and within region micronutrient deficiency and inflammation prevalences.

	Overall		Region				
			NE*	SE	SW	WC	CP
Total population sampled	4,534		1,559	988	898	627	462
Female sex (%)	2,472 (55.2)		788 (51.7)	580 (58.8)	477 (53.7)	353 (56.7)	274 (59.7)
Age in years [mean (SD)]	18.63 (16.23)		21.2 (17.4)	20.2 (17.4)	15.8 (13.6)	15.7 (13.7)	16.4 (15.9)
Vitamin B_12_ depletion	Samples	2005	754	498	455	122	176
	Cases (%)	313 (15.6)	103 (13.7)	28 (5.6)	74 (16.3)	60 (49.2)	48 (27.3)
Vitamin B_12_ deficiency	Samples	2005	754	498	455	122	176
	Cases (%)	73 (3.6)	22 (2.9)	8 (1.6)	20 (4.4)	15 (12.3)	8 (4.5)
Retinol deficiency	Samples	1780	587	476	429	1,119	169
	Cases (%)	207 (11.6)	15 (2.6)	61 (12.8)	78 (18.2)	31 (26.1)	22 (13.0)
Zinc deficiency	Samples	3,744	869	952	866	606	451
	Cases (%)	2,490 (66.5)	371 (42.7)	536 (56.3)	715 (82.6)	524 (86.5)	344 (76.3)
Ferritin-based iron deficiency	Samples	3,742	843	958	877	609	455
	Cases (%)	437 (11.7)	108 (12.8)	148 (15.4)	75 (8.6)	46 (7.6)	60 (13.2)
Transferrin receptor-based iron deficiency	Samples	3,786	876	962	879	614	455
	Cases (%)	86 (2.3)	20 (2.3)	38 (4.0)	14 (1.6)	6 (1.0)	8 (1.8)
AGP-based inflammatory status	Samples	3,827	882	969	892	622	462
	Cases (%)	918 (24.0)	183 (20.7)	243 (25.1)	238 (26.7)	164 (26.4)	90 (19.5)
CRP-based inflammatory status	Samples	3,824	881	968	891	622	462
	Cases (%)	469 (12.3)	68 (7.7)	130 (13.4)	145 (16.3)	75 (12.1)	51 (11.0)

^*^The data from the northeast used only the first time point for individuals with multiple sampled time points.

### Geographic variation of micronutrient deficiencies and inflammation

Across Madagascar, there are major regional variations in the prevalence of observed micronutrient deficiencies and inflammation for five of the seven biomarkers (Figure 1). Without accounting for the structure in the data (e.g., slight variations in demographic patterns within each region, or the nested sampling structure of individuals within households), there is a nearly 8-fold difference between the lowest and highest observed regional prevalence of vitamin B_12_, a 10-fold difference in vitamin A deficiency, a 4-fold difference in iron deficiency (based on transferrin receptor), and a ~ 2-fold difference in acute inflammation (CRP) and deficiencies of zinc and iron (based on ferritin; Figure 1). There were no major regional patterns in the prevalence of chronic inflammation (AGP). Interestingly, the severity of regional deficiencies is geographically incongruous, where locations with the highest prevalence of deficiencies in vitamin B_12_, vitamin A and zinc coincide with the lowest prevalence of iron deficiency (both ferritin and TFR) (Figure 1).

**Figure 2:**
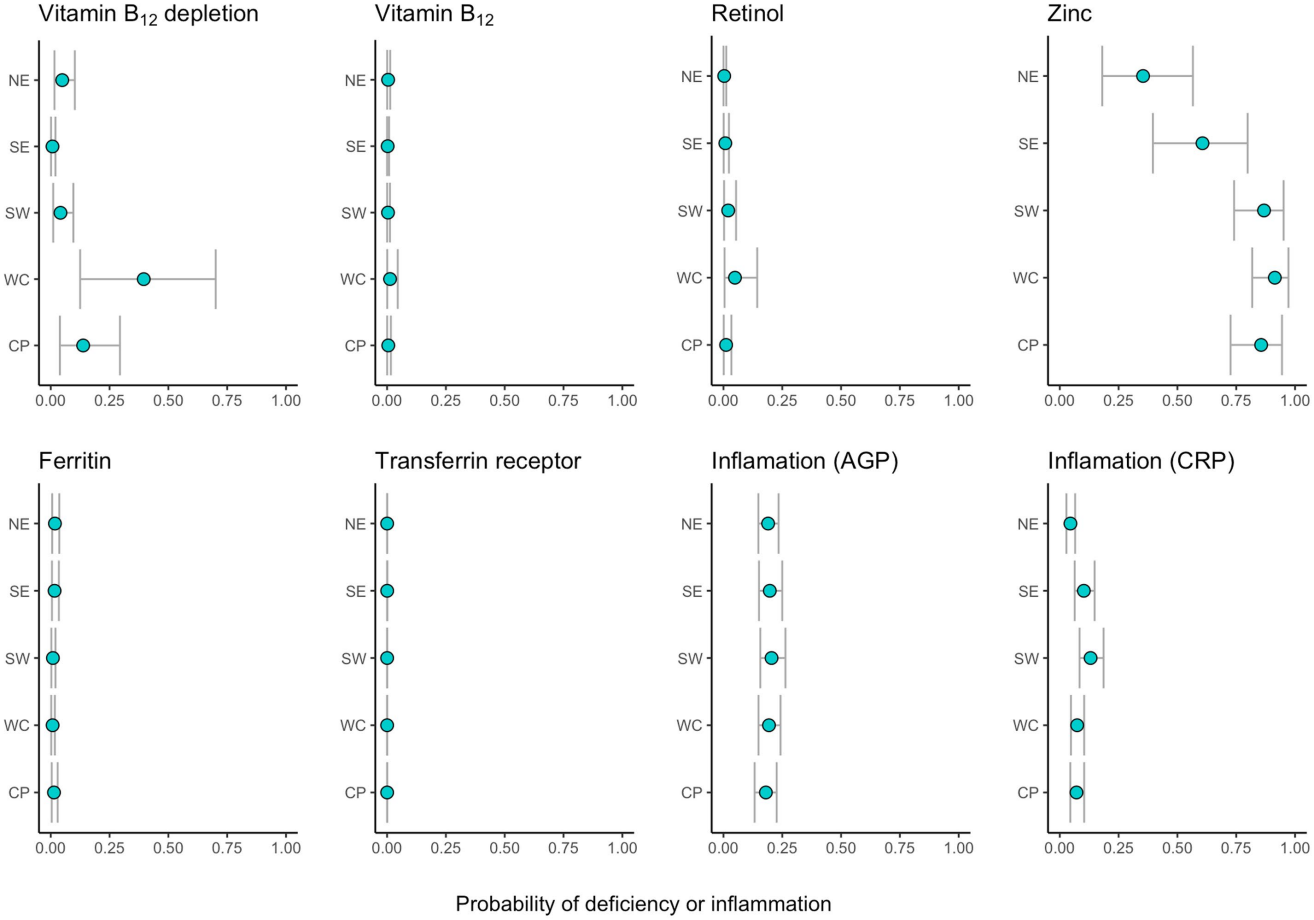
Region-specific estimated probabilities of depletion, deficiency or inflammation (for average age and using males and the dry season as a baseline). Points represent the median and intervals of the 90% uncertainty intervals for each region.

When accounting for demographic and seasonal differences among the sampled populations across regions, we modeled the probability of an average-aged individual of the male sex sampled in the dry season to control for influencing factors. We found even stronger regional differences in the probabilities of vitamin B_12_ depletion (73-fold), vitamin A deficiency (22-fold), zinc deficiency (3-fold), and acute inflammation (3-fold). Yet, the regional patterns among biomarkers remain consistent. Vitamin A and B_12_ depletion were significantly higher in the west coast (Morombe district) and lower in the southeast region (Mananjary district) in comparison to other regions (Figure 2). Zinc deficiency, while widespread, was higher in the southwest (Toliara II district) and the central plateau (Fandriana, Ambatofinandrahana, and Ambositra districts), and lower in the northeast (Maroantsetra district). Total probabilities of iron deficiency and chronic inflammation (AGP) did not vary greatly among regions when we accounted for demographic differences. However, acute inflammation (CRP) was higher in the southwest region, followed by the southeast.

Variance partitioning results of our models show regional variance is the greatest source of variation for zinc status, and the second greatest source of variation for vitamin A and B_12_ status and CRP inflammation (with individual variance estimated to be higher). For iron deficiency and AGP inflammation, other sources of variance (individual, household, or community) were estimated to be greater than region (Supplementary materials).

## Discussion

Over several years of population surveys and biological sample collections between 2013–2020 in diverse regions of rural Madagascar, we documented moderately low levels of iron deficiency (less than 15% everywhere; (31)), moderate levels of vitamin A deficiency (2.6–26.1%; (32)), moderately high levels of vitamin B_12_ depletion (5.6–49.2%; (13)), and some of the highest recorded prevalences of zinc deficiency in the world (43–87% across various regions; (33)). Furthermore, we found moderately low levels of acute inflammation (CRP) and high levels of chronic inflammation (AGP), consistent with trends of what is found globally where chronic inflammation is more prevalent than acute inflammation (34). These statistics are reflective of an overall situation of food insecurity and high infectious disease burden. Further analysis of paired dietary intake data and infectious disease status will be critical in understanding the core factors driving variation in these nutrient deficiencies, and will be presented in our team’s future research.

### Importance of micronutrient deficiencies

Each of these nutrient deficiencies has potentially significant population-wide health impacts. Zinc is an anti-oxidant, anti-inflammatory agent, and deeply influences cell-mediated immunity, being involved in the activities of more than 300 enzymes and 1,000 transcription factors (35). Zinc deficiency, in addition to being potentially fatal, is associated with an increased incidence of diarrheal disease, growth retardation, emotional disorders, infections, neurosensory disorders, and difficulties with healing (35). Given the very high prevalence of zinc deficiency and that our variance partitioning results show that region (rather than household or individual) is the greatest influence on variation in deficiency, landscape level interventions to target deficiencies are the most appropriate. Across Africa, Sierra Leone, Guinea, and Madagascar are ranked as the top 3 target countries for zinc biofortification of rice, indicating that this may be a particularly useful approach to meeting zinc requirements (36).

Vitamin A deficiency can lead to blindness, immune dysfunction, and increasing risk of infections and inflammation-driven anemia (37), while vitamin B_12_ deficiencies can lead to severe neurological and hematological impairment (38, 39). Both of these vitamin deficiencies were found to be most influenced by individual characteristics followed by regional differences, indicating that regional and individualized targeting may be most appropriate.

### Contextual importance of micronutrient deficiencies in Madagascar

Spatial, seasonal, and demographic differences in micronutrient deficiencies may be attributed to diverse social, ecological, economic, and food system factors that vary geographically, and in turn shape human diets and infectious disease exposure. Additionally, heterogenous landscapes of disease, as evidenced by differential prevalences of inflammation regionally, can also influence the spatially variable prevalences of micronutrient deficiencies (6). The Morombe region in the central western coast is the most burdened by micronutrient deficiencies while the Toliara region in southwestern Madagascar is the most affected by inflammation. Although not highly variable, iron deficiency peaks in the Mananjary region, an area known for high prevalence of malaria and intestinal parasites (40, 41).

Roughly 86% of employment in Madagascar is in the agricultural sector, predominantly subsistence farmers, producing their own food to survive (42). Subsistence activities are important because more than 90% of the population lives under the national poverty line (43). Improved agricultural production has been demonstrated to reduce poverty and food insecurity in rural Madagascar (44). However, given the harsh climates and environments in many regions, there are significant roadblocks limiting the diversity and productivity of food crops (45, 46), and even limiting the micronutrients in particular crops (47). In some regions, dietary diversity can be improved through forest and marine foraging (10, 48). In other regions, agricultural production could be improved, yet the benefits are localized because of the weak transport infrastructure that isolates local and regional food environments and reduces their capacity to provide spillover benefits to less fertile regions (49). All these factors likely contribute to Madagascar’s chronic food insecurity.

Food security in Madagascar is only expected to worsen given recent impacts of COVID-19 and natural disasters in the country. In a survey of Malagasy farmers, they indicated that their consumption patterns changed during the pandemic and that most were restricting the number of meals they were eating per day (50). Additionally, cyclones and droughts have significantly affected food production systems and worsened food security throughout the country (51, 52). Unfortunately, these climate-related impacts are complex and only expected to increase in the future, exacerbating the vulnerability of the Malagasy population (45, 53).

## Conclusion

The problem of widespread micronutrient deficiencies will not be easily solved and requires multi-sectoral intervention. Given limited budgets, spatial targeting for programming must be developed to focus on particular micronutrient deficiencies. Both health-based and food systems-based interventions are required, as nutrient deficiencies can occur in the absence of food insecurity due to high rates of infectious disease. Therefore, improving sanitation can benefit nutrition (54) and reducing the incidence of malaria, intestinal parasites, and other infectious diseases can also benefit nutritional status (55, 56).

In addition to health-based approaches, food systems must also be improved. Indigenous and traditional foods can be harnessed to improve dietary quality, including wild plants (57), animals (58), fisheries (59), and insects (60). These food systems interventions must be practiced in climate-smart and sustainable ways to ensure prolonged capacity to reduce undernutrition and micronutrient deficiencies while ensuring the sustained productivity of the environment. Our recommendation is to combine both health-based and food systems interventions into a portfolio-based approach to stem underlying causes of micronutrient deficiencies.

## Data availability statement

The datasets presented in this article are not readily available due to constraints from our ethical review. Therefore, individual biomarker data will not be publicly available. Requests to access the datasets should be directed to CG, golden@hsph.harvard.edu.

## Ethics statement

The studies involving humans were approved by the Committee on the Use of Human Subjects, Office of Human Research Administration at the Harvard T.H. Chan School of Public Health. The studies were conducted in accordance with local legislation and institutional requirements. Informed consent was obtained from adults, verbal assent was obtained from children over 12 years of age, and permission was obtained from parents or guardians of younger children. Both the HSPH IRB and the INSPC review board in Madagascar waived the requirement for written informed consent for this study, and approved the study’s consent procedures because requiring signatures was deemed culturally inappropriate for the targeted populations.

## Author contributions

CG: Conceptualization, Data curation, Funding acquisition, Investigation, Methodology, Project administration, Resources, Supervision, Writing – original draft, Writing – review & editing. JZ-M: Writing – review & editing, Data curation, Formal analysis, Methodology, Visualization. AL: Data curation, Formal analysis, Methodology, Writing – review & editing. BR: Data curation, Investigation, Methodology, Visualization, Writing – review & editing. LA: Formal analysis, Resources, Supervision, Writing – review & editing. DH: Formal analysis, Project administration, Writing – review & editing. JH: Funding acquisition, Investigation, Project administration, Resources, Writing – review & editing. CM: Funding acquisition, Writing – review & editing. HR: Data curation, Investigation, Supervision, Writing – review & editing. SS-F: Formal analysis, Project administration, Writing – review & editing. SW: Writing – review & editing, Data curation. SH: Conceptualization, Supervision, Writing – review & editing.
