# Supplementary material for: Prevalence of micronutrient deficiencies across diverse environments in rural Madagascar
Source: Front Nutr. 2024 May 16;11:1389080. doi: 10.3389/fnut.2024.1389080 (PMC11140575; doi:10.3389/fnut.2024.1389080)
Supplement: Supplementary file 1 [file Data_Sheet_1.docx]

**A: Bayesian Hierarchical Statistical Models**

In the following, we let *Y* denote biomarker deficiency or depletion status (1 for deficient/depleted, 0 for non-deficient/non-depleted) for the various nutritional biomarkers, or inflammatory status (1 for inflammation, 0 for no inflammation) for AGP and CRP. Further, *X*_1_ denotes seasonality (1 for dry season, 0 for rainy season), *X*_2_ denotes standardized age (centered at mean, scaled by twice the standard deviation), and *X*_3_ denotes gender (1 for female, 0 for male). To model deficiency/inflammatory outcomes, accounting for the inherent correlation structure induced by the sampling mechanism (observations within individuals over time, individuals within households, households within villages, villages within region) we considered the following hierarchical model:

$$logit\left( E\left( Y \right) \right)= \beta_{0}+ \beta_{1}X_{1}+ \beta_{2}X_{2}+ \beta_{3}X_{3}+ \varepsilon_{reg}+ \varepsilon_{vil}+ \varepsilon_{hh}+ \varepsilon_{ind}$$

Here, *E* represents expectation (i.e., average), $logit\left( z \right)=log \left( \frac{z}{1-z} \right)$, and the error terms $\varepsilon_{reg} \sim N(0, \sigma_{reg}^{2})$, $\varepsilon_{vil} \sim N(0, \sigma_{vil}^{2})$, $\varepsilon_{hh} \sim N(0, \sigma_{hh}^{2})$, and $\varepsilon_{ind} \sim N(0, \sigma_{ind}^{2})$ are random intercepts for region, village, household, and individual (across time), respectively. These intercept terms are drawn independently, and are shared across observations within the same level of the hierarchy, i.e., $\varepsilon_{reg}$ is constant across individuals in the same region, $\varepsilon_{vil}$ is shared across individuals in the same village, $\varepsilon_{hh}$ is shared across members of the same household, and $\varepsilon_{ind}$ is constant across observations over time within the same individual. $Finally, \sigma_{reg}^{2}, \sigma_{vil}^{2}, \sigma_{hh}^{2}, \sigma_{ind}^{2}$are the variances of the error terms.

To fit the above model for each biomarker, we used a Bayesian approach using the “brms” package [1] in R [2]. For the variance component parameters ($\sigma_{reg}^{2}, \sigma_{vil}^{2}, \sigma_{hh}^{2}, \sigma_{ind}^{2}$; Fig. S1), we used half Student-t priors with 3 degrees of freedom, and for the fixed effect parameters ($\beta$_0_, $\beta$_1,_ $\beta$_2,_  $\beta$_3;_ Fig. S2), we used flat (improper) priors (over all real numbers) [1].

The brms package fits these Bayesian models using “Stan” [3], which itself implements Hamiltonian Monte Carlo to sample from the posterior distribution. Each model was run with four chains of 3,000 iterations each, with 800 used as warm-up / burn-in, and thinning to every third iteration. If divergent transitions occurred after warm-up, the target average proposal acceptance probability (“adapt_delta” parameter in brms) was increased to 0.9, 0.99, and 0.995, until no divergent transitions remained.

To assess successful convergence, we inspected potential scale reduction factors (“Rhat” in brms output). All Rhat values in the fitted models were 1.00 or 1.01, and below the nominal threshold of 1.1 [4, 5], thus indicating successful convergence. Likewise, we assessed posterior contraction values (PCV) for the variance component parameters to ensure we were not relying too heavily on the choice of priors, and that the data were the primary drivers of evidence [6]. All PCVs observed for the main models described above were 90% or higher, with the vast majority above 95%, indicating that the data were indeed informative [6].

Finally, to assess the robustness of our findings (e.g., sensitivity to prior and model choice), we conducted sensitivity analyses (see part B). We also conducted posterior predictive checks to judge adequacy of model fits. All models fit our data well (see part C).


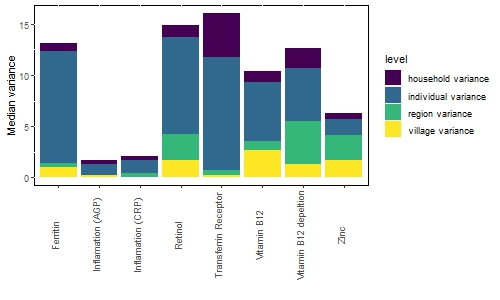


Figure S1| Variance decomposition of our deficiency and inflammation Bayesian hierarchical models. Plotted is the posterior median estimated variance ($\sigma^{2}$) for each of the random effects corresponding to region, village, household, and individuals.


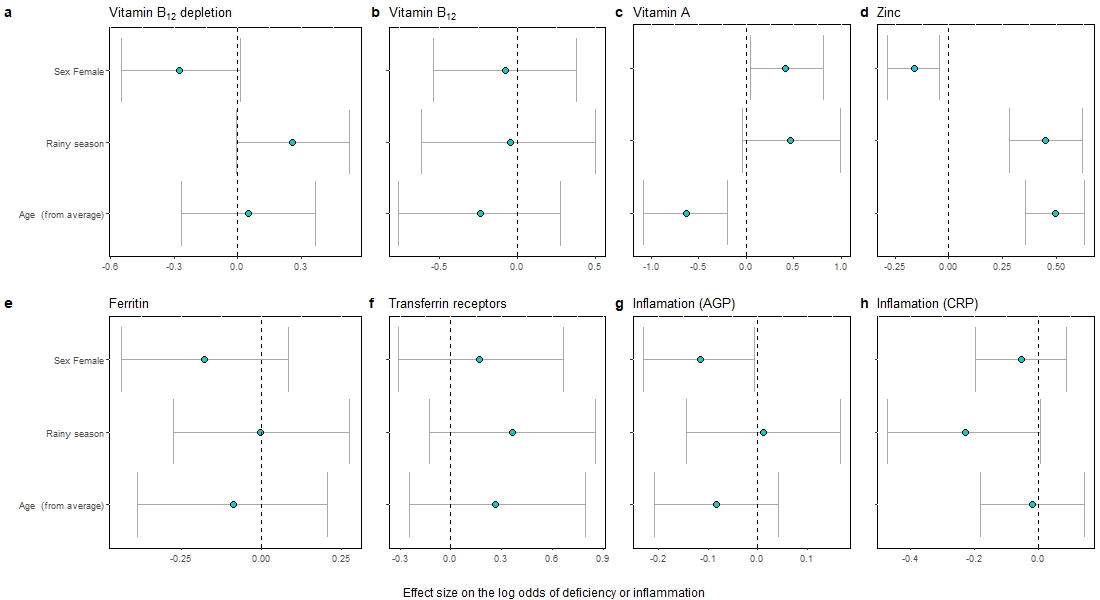


Figure S2| Estimated fixed effects in our Bayesian hierarchical model. The point is the median and the intervals represent 90% uncertainty intervals. Dashed solid line represents 0.

Supplementary Table 1: Summary of sample sizes with complete covariate information that informed the models

|  | Overall | | NE* | SE | SW | WC | CP |
| --- | --- | --- | --- | --- | --- | --- | --- |
| B_12_ Depletion | Samples | 3043 | 1811 | 495 | 448 | 117 | 172 |
| B_12_ Deficiency | Samples | 3043 | 1811 | 495 | 448 | 117 | 172 |
| Retinol Deficiency | Samples | 2740 | 1549 | 476 | 429 | 118 | 168 |
| Zinc Deficiency | Samples | 4701 | 1830 | 952 | 866 | 603 | 450 |
| Ferritin-based Iron Deficiency | Samples | 4935 | 2042 | 958 | 877 | 605 | 453 |
| Soluble transferrin receptors-based Iron Deficiency | Samples | 5023 | 2119 | 962 | 879 | 610 | 453 |
| AGP-based Inflammatory Status | Samples | 5033 | 2123 | 963 | 880 | 611 | 456 |
| CRP-based Inflammatory Status | Samples | 5029 | 2121 | 962 | 879 | 611 | 456 |

*The data from the Northeast include the repeated measures of all samples collected throughout the cohort study

**B: Sensitivity Analyses**

First, in order to assess sensitivity to the choice of prior distribution for the variance components, we ran the models (specified above) but varying the scale parameter of the half Student-t distribution (always with 3 degrees of freedom). Namely, while the main analysis had a scale parameter of 1, we ran analyses with scale parameter equal to 0.5 (i.e., a low variance prior distribution more concentrated towards 0), as well as a scale parameter equal to 5.5 (i.e., a high variance, less informative prior distribution). While such changes resulted in lower and higher PCVs, respectively, the main results did not change substantially, i.e., estimates and credible intervals for region-specific random intercepts and fixed effects changed only very slightly (Fig. S3).


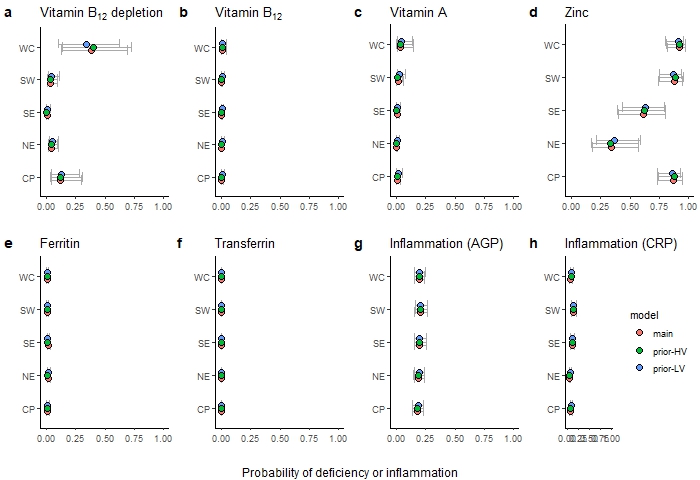


Figure S3. Region-specific estimated probabilities of deficiency (for average age and using males as a baseline) under different prior choices. Points represent the median and intervals the 90% uncertainty intervals for each region. Results are shown for the main prior choice (“main”), the high variance prior (“prior-HV”), and low variance prior (“prior-LV”).

**C: Posterior Predictive Checks**


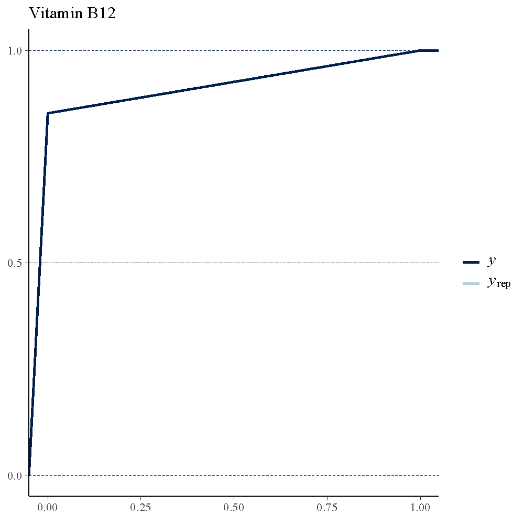


Figure S4: Posterior predictive check for the model for Vitamin B_12_ deficiency. Shown is an overlay of the observed empirical cumulative distribution function (y) in black, and cumulative distribution functions for draws from the posterior distribution (y_rep_) in light blue (where light blue lines are not apparent it means high overlap with the observed empirical cumulative distribution).


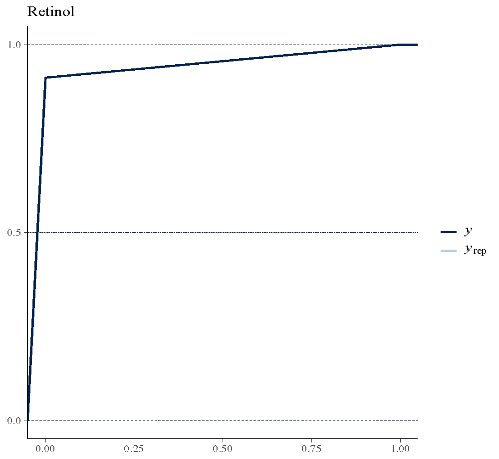


Figure S5: Posterior predictive check for the model for retinol. Shown is an overlay of the observed empirical cumulative distribution function (y) in black, and cumulative distribution functions for draws from the posterior distribution (y_rep_) in light blue.


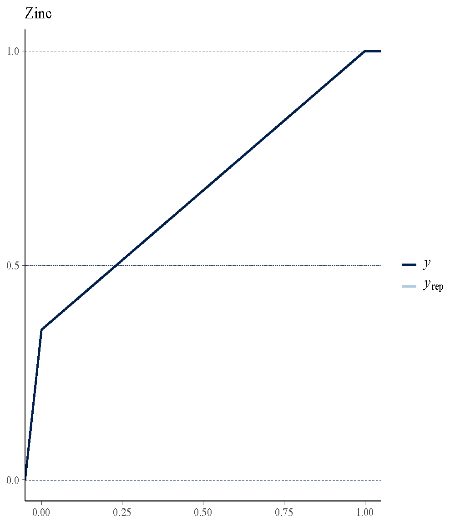


Figure S6: Posterior predictive check for the model for zinc. Shown is an overlay of the observed empirical cumulative distribution function (y) in black, and cumulative distribution functions for draws from the posterior distribution (y_rep_) in light blue.


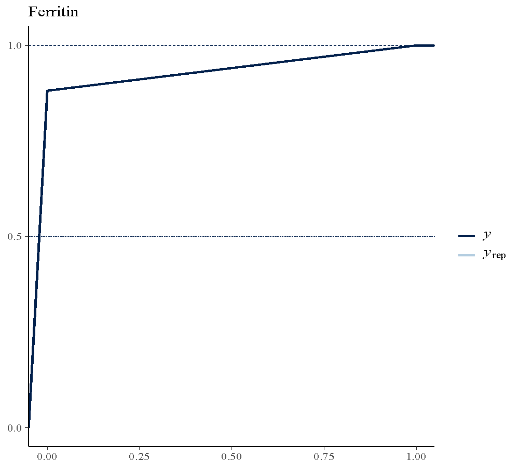


Figure S7: Posterior predictive check for the model for ferritin. Shown is an overlay of the observed empirical cumulative distribution function (y) in black, and cumulative distribution functions for draws from the posterior distribution (y_rep_) in light blue.


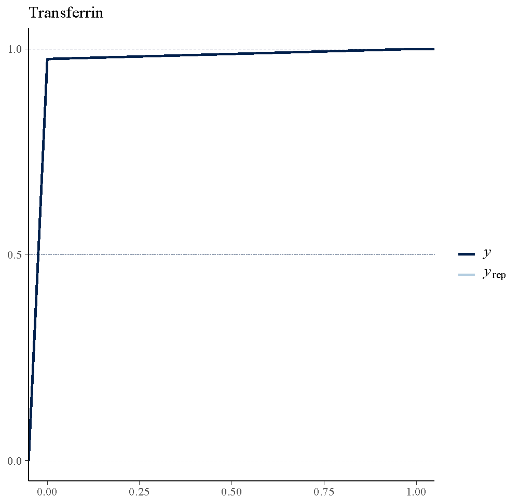


Figure S8: Posterior predictive check for the model for transferrin. Shown is an overlay of the observed empirical cumulative distribution function (y) in black, and cumulative distribution functions for draws from the posterior distribution (y_rep_) in light blue.


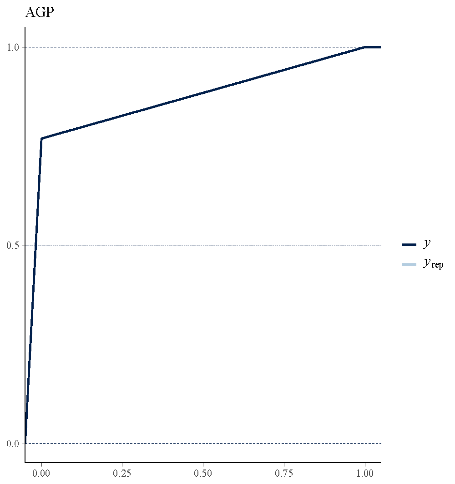


Figure S9: Posterior predictive check for the model for AGP. Shown is an overlay of the observed empirical cumulative distribution function (y) in black, and cumulative distribution functions for draws from the posterior distribution (y_rep_) in light blue.


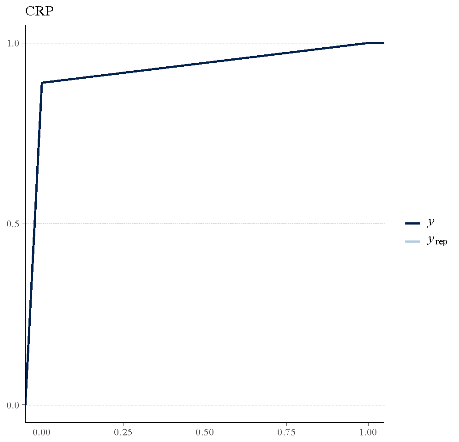


Figure S10: Posterior predictive check for the model for CRP. Shown is an overlay of the observed empirical cumulative distribution function (y) in black, and cumulative distribution functions for draws from the posterior distribution (y_rep_) in light blue.

**References**

1. Bürkner PC. brms: An R package for Bayesian multilevel models using Stan. Journal of statistical software. 2017 Aug 29;80:1-28.
2. R Core Team. R: A language and environment for statistical computing. 2021. R Foundation for Statistical Computing, Vienna, Austria. URL [https://www.R-project.org/](https://www.r-project.org/).
3. Gelman A, Lee D, Guo J. Stan: A probabilistic programming language for Bayesian inference and optimization. Journal of Educational and Behavioral Statistics. 2015 Oct;40(5):530-43.
4. Brooks SP, Gelman A. General methods for monitoring convergence of iterative simulations. Journal of computational and graphical statistics. 1998 Dec 1;7(4):434-55.
5. Gelman A, Rubin DB. Inference from iterative simulation using multiple sequences. Statistical science. 1992 Nov;7(4):457-72.
6. Schad DJ, Betancourt M, Vasishth S. Toward a principled Bayesian workflow in cognitive science. Psychological methods. 2021 Feb;26(1):103.
